# High fat diet, gut microbiome and gastrointestinal cancer

**DOI:** 10.7150/thno.56157

**Published:** 2021-04-03

**Authors:** Yao Tong, Huiru Gao, Qiuchen Qi, Xiaoyan Liu, Juan Li, Jie Gao, Peilong Li, Yunshan Wang, Lutao Du, Chuanxin Wang

**Affiliations:** 1 Department of Clinical Laboratory, The Second Hospital, Cheeloo College of Medicine, Shandong University, Jinan, Shandong, China.; 2 Shandong Engineering & Technology Research Center for Tumor Marker Detection, Jinan, Shandong, China.; 3 Shandong Provincial Clinical Medicine Research Center for Clinical Laboratory, Jinan, Shandong, China.

**Keywords:** high fat diet, gastrointestinal cancer, gut microbiome, inflammation, metabolic reprogramming

## Abstract

Gastrointestinal cancer is currently one of the main causes of cancer death, with a large number of cases and a wide range of lesioned sites. A high fat diet, as a public health problem, has been shown to be correlated with various digestive system diseases and tumors, and can accelerate the occurrence of cancer due to inflammation and altered metabolism. The gut microbiome has been the focus of research in recent years, and associated with cell damage or tumor immune microenvironment changes via direct or extra-intestinal effects; this may facilitate the occurrence and development of gastrointestinal tumors. Based on research showing that both a high fat diet and gut microbes can promote the occurrence of gastrointestinal tumors, and that a high fat diet imbalances intestinal microbes, we propose that a high fat diet drives gastrointestinal tumors by changing the composition of intestinal microbes.

## Introduction

According to 2020 statistics, 1,806,590 new cancer cases and 606,520 cancer deaths occurred in the United States, with gastrointestinal cancer being one of the leading causes of death 1. Colorectal cancer (CRC) is the most diagnosed and fatal gastrointestinal cancer. Pancreatic cancer is the second, with a five-year survival rate of only 9% in America 1. These are followed by liver, stomach and esophageal cancers. Obesity is a risk factor for gastrointestinal cancer 2, and the main cause of obesity is a high fat diet (HFD). It contains high amounts of fatty acids, but low amounts of fiber, vitamins and minerals. In recent years, with the economic development and speed-up of life, obesity has become a global health problem, leading to the prevalence of a variety of chronic diseases 3. It is estimated that, by 2025, the global obesity rate will rise to 18% for men and 21% for women 4. The increased prevalence of obesity may increase the global burden of gastrointestinal cancer.

With the development of the metagenome and macrotranscriptome in recent years, research into intestinal microorganisms has reached a new peak 5, 6. These organisms are in a dynamic state, influenced by drugs, diet, lifestyle, genetics and environmental factors 7. Researchers have found that gut microbes not only affect intestinal diseases like inflammatory bowel disease 8 and CRC 9, but also extra-intestinal disorders such as hepatic disease 10 and pancreatic disease 11. In addition, researchers have demonstrated that HFD can significantly alter the composition of gut microbes 12. We hypothesized that intestinal microorganisms may play an important role in the pathogenesis of gastrointestinal tumors induced by HFD. This review will provide a brief overview of recent studies on the relationship between HFD, gut microbes and gastrointestinal tumors, and discuss possible mechanisms by which HFD alters the composition of intestinal microorganisms to promote the development of gastrointestinal tumors.

## High fat diet promotes gastrointestinal cancers

**Figure 1:**
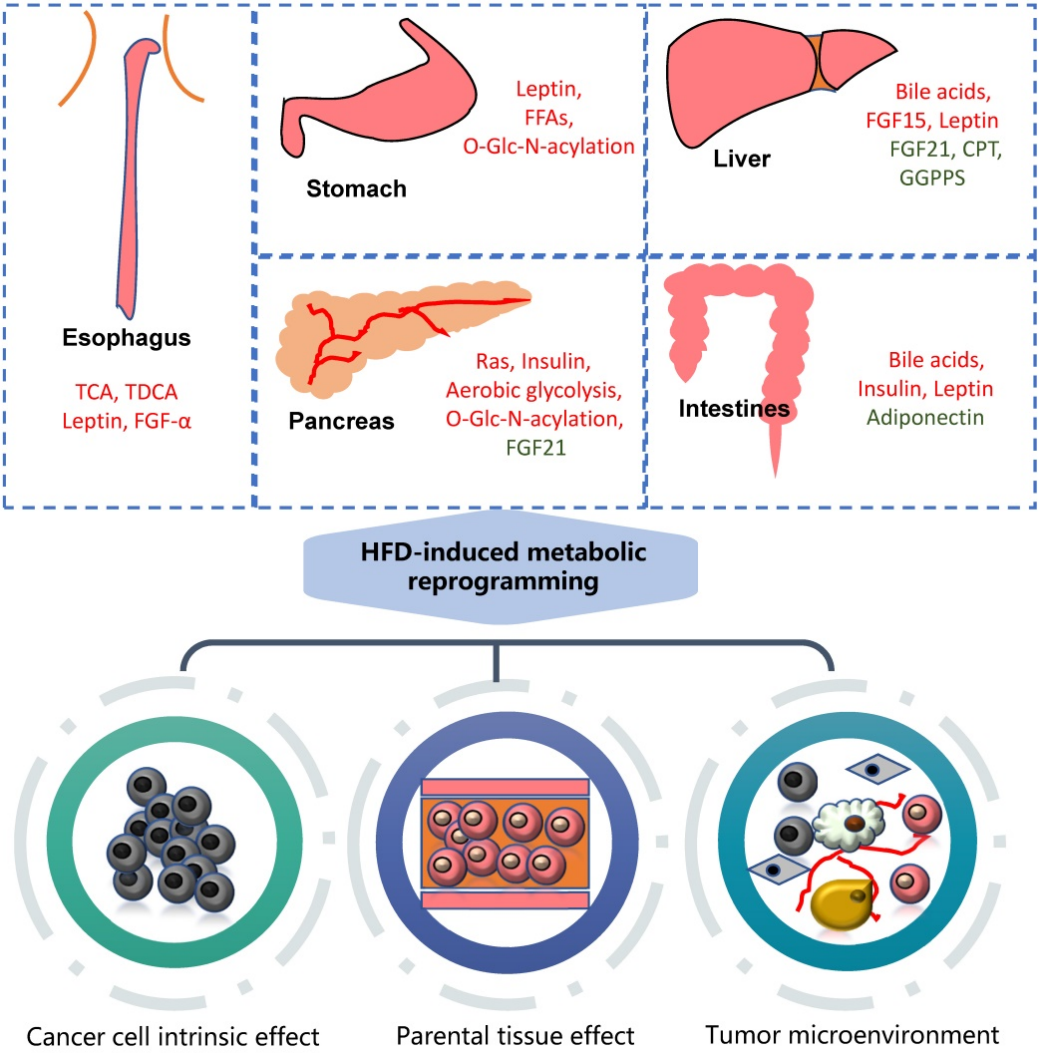
** Association of HFD with gastrointestinal cancers.** High fat diet can cause metabolic reprogramming in multiple organs and tissues of the human body with alterations in the content of various regulatory factors. It mainly acts on tumor cells themselves, nearby tissues and tumor microenvironment. The molecules marked in red are upregulated, while green downregulated. GGPPS: Geranylgeranyl diphosphate synthase.

HFD can significantly promote the occurrence and development of gastrointestinal tumors, mainly involving metabolic reprogramming and the change of various carcinogenic molecules (Figure 1).

### Esophagus cancer

As early as in 1994, it was found that mice fed HFD had a higher incidence of esophageal cancer, suggesting a relationship between HFD and esophageal cancer 13. HFD can lead to changes in the composition of bile acids of mice, especially taurocholic acid and tauroursodeoxycholic acid, leading to an increased incidence of Barrett's esophagus and esophageal cancer in mice 14. Fowler and colleagues found that esophageal adenocarcinoma in OE33 mice fed HFD had higher growth and metabolic activity and increased expression of pro-inflammatory and tumorigenic factors such as leptin, IGFBP, FGF-α in their adipose tissue, in contrast to decreased anti-inflammatory and growth inhibition molecules 15.

In clinical epidemiological studies, Ibiebele et al. found that “meat and fat” were well correlated with esophageal adenocarcinoma, gastric esophagus adenocarcinoma and esophageal squamous cell carcinoma 16. Lagergren et al. also found that a higher proportion of fat exacerbated the occurrence of esophageal carcinoma and esophageal gastric adenocarcinoma, while carbohydrates lowered the occurrence of esophageal adenocarcinoma and esophageal gastric adenocarcinoma 17.

### Gastric cancer

Many epidemiological studies have reported that dietary fat may be a risk factor for gastric cancer 18. Leptin is thought to play an important role in obesity-related gastrointestinal malignancies because of its role in angiogenesis, apoptosis, cell proliferation and cell migration 19. It has also been shown to promote mucin production and gastrointestinal tumor formation by regulating mTOR, STAT3 and ERK-dependent pathways 20, PI3K dependent pathways and MAPK dependent pathways 21. Excessive leptin and leptin signaling activation leads to gastric tumors by inhibiting suppressors of cytokine signaling 3 in gastrointestinal epithelial cells 22 as well as increased expression of ectopic molecules related to intestinal epithelium, such as intestinal mucin 2 and Paneth cell marker PLA2, as well as decreased expression of the transcription factor SRY-box transcription factor 2 and H+/K+ ATPase 23.

Arita and colleagues found that lipotoxicity associated with HFD induced precancerous lesions due to the disruption of gastric epithelial organelle homeostasis, tissue integrity, and stemness gene expression mediated by ObR signaling 24. In brief, through the up-regulation of the PI3K-Akt pathway in epithelial cells, HFD promotes β-catenin and disrupts organelle homeostasis, and can moreover up-regulate the characteristics of cancer stem cells. Various signaling molecules are expressed, such as LAMP2A, cytochrome c oxidase, LGR5 etc. Autophagy inhibition can be caused by an increase in LAMP2A, which is involved in poorly differentiated human gastric adenocarcinoma (GAC) 25.

A study found that, during 8-20 weeks of HFD feeding, mitochondrial damage was found in gastric wall cells accompanied by increased mucosal thickness. The addition of free fatty acids (FFAs) can replicate this expression and promote metagenetic changes, indicating that the lipid toxicity of FFAs induces the death of parietal cells and the occurrence of precancerous lesions. Jiang et al. found that HFD could provide sufficient energy for metastasis and increase the level of O-Glc-N-acylation, which promoted the transcriptional activation of CD36. Upregulation of CD36 leads to increased fat uptake in gastric cancer cells, forming a vicious cycle that promotes gastric cancer metastasis 26.

### Hepatic carcinoma

Metabolic reprogramming, as a key intermediate, promotes the development of tumors by connecting many pro-oncogenic molecules. Studies have confirmed that HFD can cause significantly increased hepatic retention of hydrophobic bile acids, which are significantly associated with changes in intestinal microorganisms 27. At the same time, the synthesis and transport of bile acids in the liver are disordered, leading to the release of a variety of inflammatory cytokines and the serious deposition of bile acids, and promoting the occurrence of liver cancer. In addition, a variety of molecules regulating metabolism are changed. For example, FGF21 and CPT2 were reduced in the livers of diet-induced obese mice, while FGF15 (19), IRE1α and leptin were upregulated, which were then connected with other pathological changes to promote carcinogenesis.

Reduced FGF21 is closely related to excessive proliferation, abnormal p53 and TGF-β/Smad signals 28, as well as the aberrant expression of epithelial-mesenchymal transition (EMT) and Wnt/β-catenin signals in the liver 29. CPT2, a fatty acid oxidase, was significantly down-regulated in HFD-fed mice, leading to the accumulation of acylcarnitine in hepatocellular carcinoma (HCC) tissues and serum, which synergistically inhibited the oxidation of fatty acids and activated STAT3, jointly promoting the occurrence of HCC 30. Liu and colleagues found that long-term HFD can decrease the expression of geranylgeranyl diphosphate synthase in mice 31. Liver geranylgeranyl diphosphate synthase knockout enhanced liver kinase B1 hyper-farnesylation, damaged mitochondrial function through regulating AMPK activity and promoted glycolysis. These metabolic changes led to liver inflammation, infiltration of macrophages and proinflammatory cytokines, which then promoted the liver pathological progress.

IRE1α is linked to endoplasmic reticulum stress in liver cancer and drives pathogenesis 32. On the one hand, IRE1α promotes the activation of an obesity-associated inhibitor of the NFκB pathway, leading to the production of typical pro-inflammatory cytokines such as TNF and IL-6 in the liver. On the other hand, it maintains STAT3 activation and thereby promotes hepatocyte proliferation 33. The leptin signaling pathway can activate mTOR 34 through downstream PI3K/Akt signaling, while mTOR indirectly activates eukaryotic initiation factor 4E, thus stimulating the translation of mRNA encoding proliferation and anti-apoptotic factors 35. Meanwhile, HFD can significantly increase the serum DPP4 level, promoting the cascade reaction of DPP4/CCL2/angiogenesis and the inflammatory response mediated by DPP4-regulated macrophage infiltration, all of which play a key role in HFD-related HCC progression 36.

### Pancreatic cancer

HFD can promote proliferation and inhibit abnormal cell clearance. A western diet induced excessive proliferation of pancreatic epithelial cells in mice and led to an increase in the frequency and possibility of mutations 37. HFD feeding significantly reduced the ability to remove RasV12 transformed cells, which impairs the epithelial defense against cancer 38_._

HFD can create both inflammatory and immunosuppressive tumor microenvironments. Philip et al. found that the pancreatic tissues of LSL-KRas/ela-CreERT mice fed with HFD had higher KRAS activity, fibrotic matrix, shorter survival time and higher degree of pancreatic intraepithelial neoplasms (PanIN) and pancreatic ductal adenocarcinoma (PDAC) 39. The possible mechanism is that the KRAS point mutation limits the ability of guanosine triphosphate hydrolysis, meaning Ras is continuously activated. High Ras activity can activate a positive feedback inflammatory cycle through the activation of COX-2 40, increasing the expression of inflammatory mediators, and activating and extending KRAS activity. In another study, Incio et al. found that obese mice had increased tumor weight and disseminated mesenteric peritoneal metastases as well as a fibrotic tumor microenvironment which was characterized by a lack of oxygen and hypertrophy of fat cells. The recruitment of detrimental cytokines and immune cells resulted in an increase in tumor-associated neutrophils (TANs), which mediated the activation of pancreatic stellate cells (PSCs) and the growth of tumors 41. Recently, Luo et al. found that pancreatic tissue also expressed FGF21 and its receptor proteins in addition to liver. KRAS mutation reduced the level of pancreatic FGF21, leading to increased abdominal fat accumulation, extensive inflammation, pancreatic cysts and high-grade PanIN in KC mice fed HFD 42.

Metabolic reprogramming is also involved in PDAC. For example, HFD can promote the development of pancreatic tumors by over-activating oncogenic KRAS to induce aerobic glycolysis 43. According to Chang et al., HFD can also lead to hyperinsulinemia and accelerate the formation and progression of PanIN in KRAS^G12D^-expressing mice 44. Zhang et al. found that increased endogenous insulin promotes precancerous PanIN and pancreatic cancer induced by HFD, suggesting a possible carcinogenic mechanism 45.

Hu et al. found that the mechanism of PDAC development may be related to DNA damage 46. A high sugar, high fat diet fed to C57BL/6 mice and normal pancreatic cell lines treated with high glucose *in vitro* showed significant DNA damage and increased KRAS mutation, and they also found that KRAS mutant cells had growth advantages in both normal and high glucose environments.

### Colorectal cancer

Epidemiological studies on CRC and western diets has confirmed the link between them 47. HFD promotes the occurrence and metastasis of CRC 48. Several pathways play key roles in HFD promoting CRC. The JNK pathway plays a crucial role in obesity and insulin resistance 49 and promotes carcinogenic transformation and cell proliferation. Niku et al. found that a western diet accelerated the formation of colorectal adenoma, accompanied by the heterozygous loss of the APC gene and the downregulation of the ERK1/2, AKT and mTOR signaling pathways 50. The STRA6 pathway serves as a bridge between HFD and CRC, contributing to the maintenance of CRC stem cells. HFD promotes the increase in STRA6 in tumor tissues, and STRA6 activation transduces JAK2-STAT3 signaling cascade 51. Lysine homocysteinylation, which can be increased by HFD, modifies ATR and inhibits ATR activity, and thereby disconnects the normal ATR-interacting protein connection; it moreover inhibits the downstream checkpoint kinase-1 and p53 activity of ATR, reduces the repair of DNA damage and promotes cell proliferation 52. Intestinal bile acids such as tauro-β-muricholic acid and deoxycholic acid can act as FXR antagonists, inhibit the FXR receptor, and promote the proliferation of lgr5+ tumor stem cells and DNA damage 53. HFD not only inhibits the FXR receptor, but also reduces the reabsorption of bile acid in the colonic mucosa, leading to the aggravation of toxic and side effects of bile acid on colon epithelial cells and the promotion of CRC 54. In addition, HFD can also activate the MAPK/ERK and PI3K/Akt/mTOR signaling pathways 55. In a study by Park et al., obesity caused by HFD can promote the occurrence of inflammation-related CRC, driven by the PI3K/Akt pathway and an increase in IL-12, MCP-1, IL-6 and TNF-α in the tumor microenvironment 56.

There have also been many studies on the effects of HFD on cytokines or obesity factors. Zhu and colleagues found that elevated serum levels of insulin, leptin, TNF-α, IGF1, as well as increased levels of proliferating cell nuclear antigen, COX-2, cyclinD1, β-catenin and NFκB proteins in adenoma tissues indicate that HFD promotes the formation of colonic adenomas through inflammation and metabolic abnormalities, and influences cell cycle 57. Zhang and colleagues reported that HFD can affect adipokines and cytokines in the serum and increase levels of TNF-α, IL-6 and CXCL10 58.

## HFD alters the composition of gut microbes

Many studies have demonstrated the key role of intestinal microorganisms in diseases induced by HFD, such as metabolic disorders 59 and endotoxemia 60. Therefore, we hypothesized that HFD affects the development of gastrointestinal cancer by altering the composition of gut microbes.

### High fat diet alters microbial composition regardless of duration

David et al. have shown that short-term HFD can change the composition of gut microbes 61. HFD can increase the abundance of bile-tolerant microorganisms (*Alistipes*, *Bilophila* and *Bacteroides*) and reduce the level of *Firmicutes* that metabolize plant polysaccharides (*Roseburia*, *Eubacterium rectale* and *Ruminococcus bromii*). Some metabolites, such as bile acid and metabolic enzymes (bisulfite reductase), are increased in the intestinal tract of whole-meat eaters. 61. On the other hand, Wu et al. have shown that a long-term HFD can alter the composition of gut microbes 62. They investigated 98 healthy volunteers with long-term dietary habits and performed a cross-sectional analysis between 16S rDNA sequence information and metabolites, confirming the theory of Arumugam et al. about intestinal types, i.e. that *Bacteroidetes* is highly correlated with animal proteins, various amino acids and saturated fats 63. Another study similarly showed that European children on a typical western diet, high in animal protein and fat, were dominated by the typical taxa of *Bacteroides* enterotypes, while children in Burkina Faso on a diet high in carbohydrates and low in animal protein were dominated by *Privotella* enterotypes 64.

### High fat diet leads to an increase in the F: B ratio

Recently, Bisanz et al. conducted a meta-analysis on the sequencing data of 27 animal and human studies on diet and flora and summarized the similarities between studies on the impact of HFD on intestinal flora composition. In most studies, the HFD raised the ratio of *Firmicutes* to *Bacteroidetes* 12. Moreover, the HFD group and the low fat diet group showed significant differences in the composition of intestinal flora, indicating that HFD could indeed cause changes to the intestinal flora. We have summarized the effects of HFD on intestinal microbes and the body (Table 1).

## Intestinal microbial imbalance promotes gastrointestinal neoplasms

**Figure 2:**
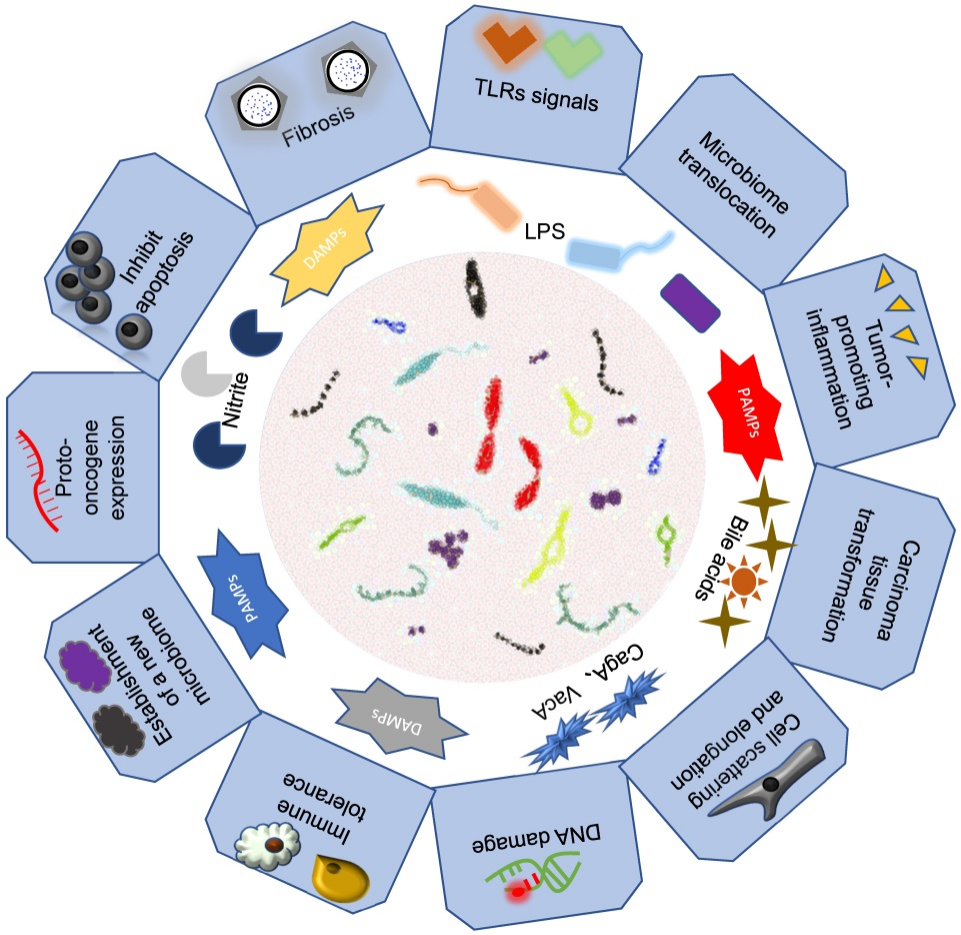
** Association of intestinal microbiomes with gastrointestinal cancers.** Intestinal microbes mainly through their bacteria or secreted metabolite components that lead to the development of gastrointestinal tumors.

Previous studies have shown that intestinal microorganisms play an important pathogenic role in some gastrointestinal tumors. We hypothesized that intestinal microorganisms may have a causal effect on gastrointestinal tumors (Figure 2).

### Esophagus cancer

Microorganisms in the esophagus are affected by oral and stomach microbes 65. *Helicobacter pylori* infection has been shown to be negatively correlated with the occurrence of esophageal cancer 66. The possible mechanism is that gastric atrophy caused by *H. pylori* infection reduces the occurrence of gastroesophageal reflux and thus reduces the occurrence of Barrett's esophagus and esophageal cancer 67. *Fusobacterium nucleatum* may lead to aggressive tumor behavior through the activation of chemokines such as CCL20 in esophageal tissues 68. Researchers have linked HFD and gut microbes to cancerous growth in Barrett's esophagus. They found that HFD accelerated esophageal dysplasia and tumor formation in L2-IL1β mice, accompanied by increased neutrophils, myeloid cells, IL-8 expression and changes in the intestinal microbiome. Under the same conditions, HFD did not accelerate the formation of esophageal tumors in germ-free mice, demonstrating that intestinal microorganisms play a key role in promoting the development of esophageal tumors in HFD mice 69. Quante and colleagues found that mice stimulated by bile acid, which is an important intestinal microbial metabolite, were more likely to accelerate the transformation of Barrett's esophagus into esophageal cancer with the expansion of lgr5+ gastric and cardiac progenitor cells 70.

### Gastric cancer

*H. pylori* induces complex inflammatory and immune responses that lead to a series of morphological events, beginning with chronic gastritis in almost all infected individuals, from atrophy to intestinal metaplasia to dysplasia and eventually to gastric cancer 71. A number of toxin proteins including CagA, VacA and outer membrane proteins are directly or indirectly involved in carcinogenesis by *Helicobacter* 72. CagA phosphorylates tyrosine by Src and Abl kinase; phospho-CagA then activates a number of host cell proteins, including Src homologous domain 2 (SH2) containing non-transmembrane phosphatases and SH2 domain-containing protein-tyrosine phosphatase, causing morphological changes such as cell scattering and elongation 73. The CagA protein of some *H. pylori* strains can stimulate the expression of interleukin by activating the transcription factor NFκB 74. It also causes DNA damage 75. Wang et al. found that hydrogen metabolism plays an important role in *H. pylori*-induced gastric cancer, and the transmembrane potential mediated by H2 oxidation catalyzed by *H. pylori* hydrogenase is involved in CagA transport and also affects DNA conversion efficiency 76. VacA results in a variety of changes in gastric epithelial cells, including vacuolation, plasma and mitochondrial membrane permeability, autophagy and apoptotic cell death 77. In addition, studies have shown that VacA and CagA can also regulate each other, affecting host cell response 78.

In addition to toxin proteins, increased secretion of pro-inflammatory cytokines, such as TNF and IL-1β, has also been shown to be closely related to tumors 79. IL-1β levels were elevated in gastric mucosa in patients with *H. pylori* infection. Recently, Koch and Muller proposed that *H. pylori* can activate the dendritic cell (DC) inflammasomes to secrete IL-1β and IL-18 80. IL-1β promotes Th1 differentiation while IL-18 promotes Treg differentiation, immune tolerance and persistent infection.

In addition, recent studies in humans have shown that the colonization of some non-*H. pylori* bacteria in the stomach affects the development of GAC 81. Gastrointestinal intraepithelial neoplasia occurred earlier in insulin-gastrin (INS-GAS) mice without *H. pylori* but colonized with intestinal flora than in sterile mice 82. Similar results were obtained in the K19-WNT1/C2mE GAC model 83. In the other study, Gantuya et al. tested *H. pylori* negative gastritis, *H. pylori* positive gastritis and *H. pylori* negative non-gastritis groups. *Streptococcus spp.*, *Haemophilus parainfluenzae* and *Treponema spp.* were possible pathogens 84.

*H. pylori* can also affect other microbiomes with an impact on gastric cancer. For example, a study showed that compared with the uninfected stomach, *H. pylori* infection increased *Proteobacteria*, *Spirochetes* and *Acidobacteria*, while it decreased the relative abundance of *Actinobacteria*, *Bacteroidetes* and *Firmicutes* 85. Lertpiriyapong et al. proposed that restricted altered Schaedler's flora (including *Clostridium* and *Lactobacillus*) alone was sufficient to promote the gastric pathogenesis of INS-GAS mice, while *H. pylori* accelerated the occurrence and progression of gastrointestinal intraepithelial neoplasia in INS-GAS mice, and showed severe inflammation in serum and local gastric tissues 86. High expression of TNF-α, IL-17, CCL2 (MCP-1) and IL-11 were found to be positively correlated with *H. pylori* infection and gastric atrophy 83. The changes in the stomach caused by chronic *H. pylori* infection that reduce the secretion of gastric acid enable the successful establishment of a new microbiome that promotes malignant transformation 87.

Recently, Inasco et al. performed studies on gastric cancer and proposed that lactic acid bacteria (LAB) can affect gastric cancer through various mechanisms 88. LAB increases thorough the development of GAC, rather than specimen contamination and dead bacteria. Lactic acid is a fuel source for cancer cells and promotes inflammation, angiogenesis, metastasis and EMT 89. LAB is a inducer of reactive oxygen species (ROS) 90, which has been shown to cause DNA damage in cells 91. In addition, LAB has been shown to reduce nitrate to nitrite, thus forming a large number of N-nitroso compounds, which promote mutation, angiogenesis, proto-oncogene expression and inhibit apoptosis 92. When LAB were grown on atrophic gastric mucosa, it had the ability to induce immune tolerance 93, which was conducive to the colonization of other important carcinogenic pathological organisms including *Veillonella*, *Prevotella*, *Fusobacterium* and *Leptotrichia* 94.

### Hepatic carcinoma

There is increasing evidence that the pathophysiology and treatment of various liver diseases may be strongly influenced by the gut microbiota 95. Intestinal microflora imbalance is associated with liver diseases such as lipid accumulation, stellate cell activation, immune cell recruitment and cancer development 96. In addition, increased bacterial translocations and disorders have been observed in the early stages of HCC and contribute to the progression of inflammation, fibrosis and cirrhosis 97. The evidence that intestinal clearance with antibiotics can significantly reduce the incidence of HCC further confirms the close relationship between microorganisms and HCC 98.

Animal model studies have shown that liver inflammation is correlated with intestinal mucosal barrier dysfunction, suggesting that bacterial translocation and imbalance caused by intestinal mucosal barrier dysfunction affect the occurrence of non-alcoholic fatty liver disease, non-alcoholic steatohepatitis (NASH) and progression to HCC 99. Firstly, bacteria components such as lipopolysaccharide (LPS) can promote the activation of TLR signals, and the release of inflammatory cytokines can promote the progress of NASH 100. IL-6 activates JAK-STAT3 pathway 101 and IL-1β activates PI3K-MDM2 to negatively regulate the p53 pathway, thereby increasing DNA-damaged cell survival 102. Secondly, the bacteria can produce ethanol and increase it in patients with NASH 103. Ethanol is not only a hepatotoxin, but also a known carcinogen that affects the development of HCC 104. Thirdly, gut bacteria can affect bile acid metabolism 105. Long-term exposure to excessive bile acids in the liver leads to increased oxidative stress, resulting in DNA damage, mitochondrial damage and damage to the cell membrane of liver cells, finally activating Ras and NFκB to induce liver cancer 106. Another mechanism controlling liver tumor growth in bile acid metabolism is the regulation of liver CXCL16 expression and CXCL16-mediated natural killer T cell recruitment 10.

### Pancreatic cancer

In recent years, many studies have found that the occurrence and development of pancreatic cancer are correlated with intestinal microorganisms, and the microorganisms in the pancreas are affected by intestinal microorganisms. It was found that the *Firmicutes*/*Bacteroidetes* ratio was significantly reduced in the feces of pancreatic cancer patients 107. Mendez et al. found that, compared with one-month KPC mice, the abundance of *Firmicutes*, *Deferribacteres*, *Actinobacteria* and *Proteobacteria* decreased significantly. Moreover, the metabolism of the microbiota changed from dominant energy metabolism to enhanced polyamine and lipid metabolism 108. Ren and colleagues found that the α diversity of pancreatic cancer patients reduced. In the intestinal microorganisms of pancreatic cancer patients, there is an increase in some potential pathogens and lipid-producing bacteria, as well as a decrease in some probiotics and butyrate producing bacteria 109. In addition, there are significant differences in the microbial composition of the duodenal mucosa 110, intraductal papillary mucinous tumor fluid 111 and bile 112 between patients and normal controls. In recent years, some researchers have focused on the correlation between pancreatic microbes and pancreatic cancer. Mitsuhashi and colleagues found that, compared with the* Fusobacterium* negative group, the cancer-specific mortality observed in the positive group was significantly higher, and the presence of *Clostridium* was significantly correlated with a poor prognosis of pancreatic cancer 113. In mice, the intestinal microorganisms of PDAC patients affected the microorganisms and volume of pancreatic tumors, as the tumors of mice transplanted with fecal bacteria from patients with a short survival time were larger, indicating that intestinal microorganisms obviously influence the microorganisms in the pancreas and the development of pancreatic cancer 114.

Traditionally, the pancreas has been considered a sterile environment 115. However, in recent years, research has shown that the pancreas contains microbes that can be affected by gut microbes 114. How microbes get into the pancreas remains a matter of debate. Several possible mechanisms may be involved 116. One is the oral route. The microorganisms in the upper digestive tract such as the oral cavity, esophagus, stomach, duodenum or biliary tract can enter the pancreatic parenchyma through the pancreatic duct 117. Secondly, microorganisms may be transferred to mesenteric lymph nodes through mesenteric lymphatic drainage, and then enter the pancreatic parenthesis through immune cell trafficking. Thirdly, anatomically, because mesenteric vein drainage enters the liver through the pancreas, microorganisms can also transfer through the wall of the colon. As a result of environmental factors, the intestinal flora is imbalanced, increasing intestinal permeability and causing microorganisms to enter the blood and reach the pancreas through the blood circulation 118.

Regarding the mechanism of the influence of microorganisms on the occurrence of pancreatic cancer, Thomas and colleagues found that there is an extra-intestinal, long-distance interaction between intestinal microorganisms and the pancreas that can promote the development of pancreatic cancer and influence transcriptional changes in pancreatic cancer xenografts and the infiltration of innate immune cells 119. Pushalkar and colleagues orally fed fluorescently labeled *Enterococcus faecalis* and GFP-labeled *Escherichia coli* into wild-type mice. They found that the bacteria could migrate to the pancreas 120. They also found that pancreatic cancer microbes inhibit innate and adaptive immunity within the tumor. In the presence of pancreatic cancer microorganisms, immunosuppressive CD206+ M2-like tumor-associated macrophages (TAMs) increased, M1-like TAMs decreased, CD4+ T cell differentiation to Th1 cells decreased and cytotoxic CD8+ T cells decreased. Activation of pattern recognition receptors in the tumor microenvironment, especially TLR2, TLR4 and TLR5, can accelerate the growth of pancreatic cancer and enhance innate and adaptive immunosuppression 120. Aykut et al. found that orally fed microbes could be transferred from the intestinal lumen to the pancreas within 30 minutes 11. *Malassezia* can promote the occurrence of pancreatic cancer. Meanwhile, Sethi et al. found that the removal of microorganisms significantly inhibited pancreatic tumor volume and reduced the risk of pancreatic cancer liver metastasis. However, in Rag1 knockout mice lacking mature T and B lymphocytes, the tumor inhibitory effect was significantly eliminated after the removal of microorganisms, suggesting that the tumor inhibition effect may be mediated by the immune system 121.

### Colorectal cancer

In clinical studies, gut microbes have been shown to be directly associated with the development of CRC. Yu et al. found that *F. nucleatum*, *Peptostreptococcus stomatis*, *Parvimonas micra* and *Solobacterium moorei* were significantly correlated with the occurrence of CRC 122. In a case-control study, the researchers found that antibiotics play a promoting role in pancreatic cancer by comparing 28,980 cases of CRC patients and 137,077 healthy controls 123. Cao et al. found that exposure to antibiotics early in life was significantly positively associated with the risk of colorectal adenoma after age 60, suggesting a potential mediating role of the intestinal microbiota in carcinogenesis 124. In addition, adenoma is an important precancerous lesion in CRC and the intestinal microbial diversity of adenoma patients is significantly reduced 125. In the classification analysis, *Ruminococcaceae*, *Clostridiaceae* and *Lachnospiracea* in the feces of patients significantly decreased, while *Bacillus* and *Gammaproteobacteria* significantly increased. In addition to bacteria, *Temperate bacteriophages* have also been shown to be associated with the occurrence and progression of CRC, as they interact with the host bacterial community and accelerate the occurrence of CRC by changing its composition 126. In terms of intestinal microbial functions, the production of LPS, polyamine synthesis, butyrate metabolism and oxidative phosphorylation are related to the occurrence and development of CRC. For example, *Mucispirillum schaedleri* can promote the production of LPS and aggravate the inflammation. *Lachnospiraceae bacterium A4* reduces butyrate generation and promotes the formation of cancers 127.

The role of some specific microbial communities in the development of CRC has been clarified, such as *F. nucleatum*, *E. coli* and *Bacteroides fragilis*. *F. nucleatum* is one of the common bacterial species in CRC 128. In clinical practice, it has been shown to be related to the occurrence of CRC, as well as metastasis and prognosis 129. It can bind with human TIGIT via fibroblast activation protein 2, which is an inhibitory receptor on all human NK cells and various T cells; this inhibits the cytotoxicity and T cell activity of NK cells against tumor cells and protects tumor cells from attack by immune cells 130. Fibroblast activation protein 2 can also bind to D-galactose-B (1-3)-N-acetyl-D-galactosamine on the surface of CRC cells, promoting the development and metastasis of CRC 131. In CRC cells, *F. nucleatum* infections can activate the TLR4 and MyD88 signaling pathways, leading to the activation of NFκB and increased miR21 expression. It can also increase the expression of anoctamin-1 in CRC cells and prevent apoptosis 132. The specific *Fusobacterium* adhesin A (FadA) of *F. nucleatum* can bind to E-cadherin, promoting intracellular annexin A1 expression, forming a FadA/E-cadherin/annexin A1/β-catenin tetramer, thereby activating the β-catenin pathway and promoting CRC cell proliferation 133. In addition, *F. nucleatum* was shown to up-regulate CARD3, activate the autophagy pathway in cells, and then promote the metastasis of CRC 134. For *E. coli*, researchers have known for years that it promotes CRC 135. Colibactin-producing *E. coli* infection can induce the phosphorylation of H2AX (a marker of DNA double-strand break), transient DNA damage, incomplete DNA repair, chromosomal aberrations and increased cell division 136. *E. coli* infection can also promote the occurrence and development of CRC by activating the P38 MAPK pathway in macrophages to up-regulate COX-2 expression 137. In addition,* B2 E. coli* isolated from CRC tissues was found to increase the invasion of multinucleated cells and the formation of crypt abscess and promote the expression of proliferative nuclear antigen in epithelial cells 138. It has been shown that genotoxic pks+ *E. coli* resulted in a distinct mutational signature to promote CRC 139. *Enterotoxigenic Bacteroides fragilis* (ETBF)can activate the Stat3 pathway in colonic epithelial cells through *Bacteroides fragilis* toxin (BFT), combine with extracellular IL-17 to activate the NFκB signaling pathway via IL17R, and trigger the expression of chemokines such as CXCL1 which can lead to the accumulation of myeloid cells in the distal colon and lead to cancer 140. Thiele Orberg et al. found that ETBF tumorigenesis required the synergistic effect of BFT and IL-17 driven inflammatory response to coordinate the recruitment of myeloid cells to tumor microenvironment and activate the immunosuppressive myeloid-derived suppressor cells (MDSCs), especially iNOS^hi^MO-MDSCs 141. BFT can also up-regulate the production of spermine oxidase in colon epithelial cells, resulting in increased ROS and DNA damage. Spermine oxidase is a source of ROS induced by bacteria, which is directly related to tumorigenesis 142. ETBF can lead to colonic mucus degradation and thereby increase the adhesion of *E. coli*, promoting colibactin-induced DNA damage to colonic epithelial cells 143. In addition to these three bacteria, it was found that *Campylobacter jejuni* 81-176 clinically isolated from humans could activate the cytolethal distending toxin (CDT) B gene, promote the production of the CDT subunit CDTB, and thereby play the role of gene toxin and promote the occurrence of CRC 144. Infection of *C. jejuni* 81-176 also changes the expression of host genes, up-regulates the PPAR signaling and calcium signaling pathways and alters the intestinal microbial composition and gene transcription of the host. *Lysinibacillus sphaericus* in the gut degrades aspirin and reduces its chemopreventive effects regarding CRC in mice 145.

Intestinal microbial imbalance can promote the occurrence of colorectal inflammation and then lead to CRC. For example, under normal circumstances, symbiotic fungi in the intestine can be recognized by C-type lectin receptors, activating downstream SYK and CARD9, which in turn mediates the activation of inflammasomes and promotes the maturation of IL-18 and the anti-tumor T cell response, thereby limiting the development of CRC. Imbalanced changes in intestinal microorganisms may inhibit the anti-tumor effect of the SYK-CARD9 axis 146. In addition, fungal dysregulation increases and accumulates the MDSCs in the lamina propria of the colon, thereby promoting the development of CRC, while CARD9 can limit the accumulation of MDSCs and inhibit cancer development 147. In CRC, several flagellar microorganisms such as *Proteobacteria* increase in intestinal microorganisms. Flagellin is an important component of microorganisms, which can increase the secretion of IL-6 and CCL2/MCP-1 mRNA expression by C26 CRC cells, reduce caspase-1 activity and active oxygen production, thereby increasing cytotoxicity, leading to an increase in the inflammatory response, which plays a role in promoting cancer 148.

In addition, intestinal microbial metabolites can also promote CRC. For example, short-chain fatty acids (SCFAs) are products of microbial catabolism of dietary fiber in the colon and cecum, including acetate, propionate and butyrate 149, which can be combined with Ffar2 expressed on ILC3s 150. Through binding with Ffar2, acetate increases the number of ILC3s in the colon, and propionate promotes the number of IL-22+ ILC3s, reducing inflammation in the colorectum 151. The down-regulation of Ffar2 leads to increased tumor bacterial load, promotes the failure of CD8+ T cells, excessively activates DCs and promotes the occurrence of colorectal tumors 152 (Table 2).

## High fat diet promotes the development of gastrointestinal tumors by changing intestinal microbes

### Esophageal cancer

Changes in intestinal microbes due to HFD will cause an increase in immature myeloid cells and neutrophils of esophagus tissues. The progenitor cells, i.e. lgr5+ cells, expand into the esophagus accompanied by chronic inflammation, thereby causing metaplasia and dysplasia Meanwhile, the intestinal microbiota promotes the development of esophageal cancer by activating IL-8 via TLR signaling, which has an impact on the immune system and activates granulocytic myeloid cells 69. Hydrogen sulfide (H_2_S) has been shown to promote the occurrence of various cancers, which is a metabolite of intestinal microorganisms 153. Studies have found that HFD can induce gut microorganisms such as *Desulfovibrio spp.* and *Clostridium lavalense* to produce carcinogenic H_2_S 154, while H_2_S in the blood circulation is mainly microbial origin, and the amount of H_2_S in the blood of germ-free mice is very low 155. After the increased H_2_S reaches the esophageal tissue through the blood circulation, by up-regulating HSP90, it promotes the proliferation, anti-apoptosis, angiogenesis and migration of esophageal cancer cells 156.

### Gastric cancer

A recent study found that gastric leptin signal regulates gastric flora and intestinal metaplasia of gastric mucosa. Stomach flora transplantation in HFD group induces intestinal metaplasia in recipient mice, indicating that microbial changes promote intestinal metaplasia 157. In this study, *Lactobacillales* increased in the stomach of mice fed HFD, while *Bifidobacteriales* decreased. *Lactobacillus* converts lactose to lactic acid, acidifying the surface of the gastric mucosa 158. Lactic acid can serve as a fuel source for cancer cells and promote inflammation, angiogenesis, metastasis and EMT 159. LAB is also an inducer of ROS 90, causing DNA damage to cells. Some LAB can reduce nitrate to nitrite 160, which promote mutation, angiogenesis, proto-oncogene expression, and inhibit apoptosis 92. It has the ability to induce immune tolerance 93, which is beneficial to colonize other important carcinogenic pathological organisms including *Veillonella*, *Prevotella*, *Fusobacterium*, *Leptotrichia* and promote tumorigenesis in various ways 161, but this still needs further study.

He et al. found that, after 12 weeks of HFD, the gastric flora was abnormal and the diversity of the flora decreased 162. Besides, HFD-induced overgrowth of *Enterobacteriaceae* can increase endotoxin production, thereby further triggering chronic inflammation and accelerating obesity 163. The research of Xiao et al. showed that *Desulfovibrionaceae* may be an important group of endotoxin producers that can produce LPS and induce chronic inflammation and metabolic endotoxemia 164, which can be observed in the stomach of mice fed with HFD. LPS stimulation induces the expression of CXCR7 in gastric cancer and promotes the proliferation and migration of gastric cancer cells through the TLR4/MD-2 signaling pathway 165. In addition, TLR4/CD74/MIF can also promote cell proliferation 166. TLR4 signal activation can also promote gastric cancer cell proliferation by generating mROS 167; LPS-NFκB-PD-L1 axis can also affect immune escape 168. Meanwhile, *Desulfovibrionaceae* reduces sulfate to H_2_S and destroys the intestinal barrier 169. H_2_S upregulates the expression of the fatty acid receptor CD36 in gastric cancer cells and directly activates CD36, triggering lipid metabolism reprogramming and thereby promoting gastric cancer metastasis 170.

### Hepatic carcinoma

There exist some scholars who have done research on HFD promoting HCC through intestinal microorganisms. Xie et al. found in a mouse model of NASH-HCC induced by streptozotocin and HFD that hydrophobic bile acids significantly increased intrahepatic retention, and these are significantly associated with changes in intestinal microbes 27. At the same time, the dysregulation of bile acids synthesis and transport in the liver leads to the release of various inflammatory cytokines and the severe accumulation of bile acids, which promotes the occurrence of liver cancer. Long-term mechanical exposure to excessive liver bile acids leads to increased oxidative stress, which can lead to DNA damage, mitochondrial damage and destruction of liver cell membranes, and activate Ras and NFκB 106. The activated NFκB is transferred to the nucleus, and promotes the expression of genes encode proinflammatory cytokines, such as TNF-α, IL-1β and IL-6. IL-6 can activate the JAK-STAT3 pathway, leading to reduced apoptosis and HCC progression 101. IL-1β activates PI3K-MDM2 and negatively regulates the p53 pathway, thus increasing the survival of DNA-damaged cells, which may lead to the development of liver cancer 102. Disturbance of bile acids on cell membranes can also activate intracellular PLA2, allowing cell membranes to release arachidonic acid, which is metabolized by lipoxygenase and COX in liver cells and goes on to generate ROS. Reactive oxygen can directly activate NFκB or induce direct DNA damage to cells, which may lead to HCC 171. Another mechanism for controlling liver tumor growth in bile acid metabolism is that secondary bile acids inhibit liver CXCL16 expression and CXCL16-mediated natural killer T cell recruitment to suppress antitumor immunity 10.

Similarly, a study found that HFD can cause excessive growth of Gram-positive bacteria in the intestine and increase the level of deoxycholic acid in the liver. Moreover, the aging-related hepatic stellate cell secretory phenotype produces various inflammatory and tumor-promoting factors, thereby promoting the development of HCC in mice exposed to the chemical carcinogen DMBA 98. Deoxycholic acid and lipoteichoic acid in the liver are excessively displaced, and recognized by the TLR2 receptor of hepatic atellate cells to upregulate the senescence associated secretory phenotype (SASP) factors and COX-2, and then produce PGE2 to suppress anti-tumor immunity through EP4 172. In addition, Xie et al. used the NASH-HCC C57BL/6J mouse model induced by streptozotocin and HFD to observe the changes in intestinal microbes. They found that *Atopobium spp.*, *Bacteroides spp.*, *Bacteroides vulgatus*, *Bacteroides acidifaciens*, *Bacteroides uniformis*, *Clostridium cocleatum*, *Clostridium xylanolyticum* and *Desulfovibrio spp.* were significantly increased in model mice, positively correlated with LPS levels and the liver disease pathological progress 173; the interaction between LPS and TLR is a possible mechanism.

In addition to the pathways mentioned above, we have also investigated other pathways. For example, while HFD can cause abnormal accumulation of bile acids under the action of microorganisms, and Cui et al. found that the liver FGF15 (19)/FGFR4 signal is significantly enhanced by HFD, we infer that bile acids enhance FGF15 (19)/FGFR4 and then activate EMT and the Wnt/β-catenin pathway, leading to the cancerous conversion of liver cancer 174. In addition, HFD can reduce the proportion of *Akkermansia muciniphila* 175 and increase lipotoxicity 176; we assume that HFD reduces the content of this flora, increases the damage due to lipotoxicity and activates the UPR signal conducting molecule, IRE1α 32, 177. In addition, intestinal microbes may be related to the level of Dpp4 178, so the Dpp4 pathway we mentioned above is likely to involve intestinal microbes.

### Pancreatic cancer

We propose several mechanisms to support the hypothesis that HFD promotes the development of pancreatic cancer by affecting the composition of intestinal microbes. A number of studies have shown that HFD can affect human blood glucose levels through the intestinal flora. For example, HFD can cause the decline of *Lactobacillus* in the upper small intestine. This flora is related to the expression of sodium glucose cotransporter-1 in the small intestine and the secretion of GLP 1 179. Therefore, we speculate that HFD results in a reduced ability to regulate blood sugar and insulin resistance, leading to hyperglycemia. High glucose can increase fructose-6-phosphate/glucose-6-phosphate in pancreatic cells, which upregulates the level of glycosylation modification of O-GlcNAc, destroys functional formation of the ribonucleotide reductase complex, depletes the dNTP pool and triggers DNA replication pressure. Collectively, this leads to genome instability, increased gene mutations and a higher risk of pancreatic cancer 46.

HFD proliferates pro-inflammatory flora such as *Bilophila wadsworthia*, promotes the translocation of LPS to pancreatic tissues and accelerates the occurrence of pancreatic cancer through inflammation. We speculate that HFD exacerbates intestinal barrier dysfunction, resulting in the accumulation of flora and LPS, which may translocate into pancreatic tissue through the destroyed intestinal barrier and stimulate an inflammatory positive feedback loop to synergistically enhance Ras to promote pancreatic cancer. Since NFκB is a common targeting molecule downstream of Ras, we surmise that translocated LPS stimulates TLRs of pancreatic tissue and stimulates the downstream NFκB pathway, enables downstream COX-2 and promotes a positive inflammatory feedback loop through the production of PGE2 180, thereby sustaining inflammatory response and fibrosis activation to promote tumorigenesis. COX-2 signaling can also activate glucose-regulated protein 78 through the cAMP/PKA pathway via the EP receptor of epithelial cells 181, then activate the PI3K/AKT pathway, and play an important role in tumor development in various ways 182.

It has been pointed out that complement activation in the tumor microenvironment can promote tumor immune escape, proliferation, and metastasis by maintaining T cell immunosuppression and chronic inflammation 183. Meanwhile, a variety of complement-derived effectors and downstream signaling molecules participate in these processes, including the anchoring and proliferation of tumor cells, as well as tumor-related angiogenesis, matrix remodeling, migration, tissue invasion and metastasis 184. Based on a study by Aykut et al. 11, we speculate that HFD can promote the translocation or migration of fungi like *Malassezia spp.* to pancreatic tissue, which combines with mannose binding lectin (MBL) and stimulates the complement cascade by the lectin pathway, leading to the release of C3a, C5a and other substances. C3aR-mediated signal transduction triggers the release of neutrophil extracellular traps and the polarization of neutrophils to the tumorigenic phenotype 185, while C5a can recruit MDSCs into solid tumors. MDSCs suppress effector T cells by exerting a powerful immunosuppressive effect by depriving amino acids, producing nitric oxide and ROS, up-regulating the expression of PD-L1 and secreting angiogenic factors 186, forming an immunosuppressive inflammatory microenvironment. HFD increases the abundance of *F. nucleatum* in the intestine, which may transfer from the intestine to the pancreas through mesenteric vein drainage, and promote the occurrence of pancreatic cancer. In HFD-fed mice, the abundance of *Fusobacterium* increased significantly 187. As mentioned above, intestinal microorganisms may enter the pancreas through mesenteric vein drainage due to the disordered intestinal barrier. The fibroblast activation protein 2 protein of *F. nucleatum* combines with TIGIT in the pancreas 130, regulates the tumor microenvironment, reduces CD3+ T cells and CD4+ T cells, increases M2-like TAMs, suppresses the attack of the immune system on tumor cells, and promotes the development of pancreatic cancer 188. Since HFD can promote pancreatic fat tissue accumulation and this pancreatic fat infiltration is significantly associated with the occurrence of pancreatic cancer 189, an excessive diet in healthy men has been shown to increase plasma LPS levels. LPS activates pro-caspase-11, corresponding to human caspase-4/5 in mice through an atypical reaction independent of TLR4, and induces an immune response 190. Therefore, we also speculate that the activated caspase-11 (caspase-4/5) controls the assembly of apoptotic speck-associate protein by NLRP3 191, and then activates capase-1 to release IL-1β and IL-18 192. IL-1β plays an important joint amplification effect between cancer-associated adipocytes, TANs and PSCs 41.

### Colorectal cancer

Regarding the pathogenesis of CRC, some scholars have found that intestinal microbes can serve as a bridge in the process of HFD promoting CRC development, connecting the causal relationship between the two.

Schulz and colleagues found that HFD feeding promoted the formation of tumors in the small intestine of mice, along with changes in the composition of intestinal microbes. This transformation of intestinal microbes is related to the reduction of host antibacterial defenses mediated by Paneth cells, which reduces the recruitment of DCs and the expression of MHC class II molecules in intestinal associated lymphoid tissues. In terms of microbial metabolites, HFD can cause a significant reduction of SCFAs in the small intestine and feces 193. Gaines et al. found that the formation of colorectal tumors is relevant with collagenase-producing microbes in the intestine such as *Enterococcus faecalis*, *Proteus mirabilis* and *Candida parapsilosis*. The colonization of these bacteria at the anastomosis destroys the healing intestine, resulting in increased permeability and transmigration of cancer cells, which promotes the recurrence of CRC. HFD increased collagenase-producing microbes 194, accelerating this effect. MCP-1 is a cell factor that acts through its receptor CCR2 to recruit the circulating leukocytes to the site of inflammation 195. The intestinal microbial imbalance caused by HFD mediates the activation of the MCP-1/CCR2 axis, recruits monocytes to the tumor microenvironment and promotes polarization to TAMs, thereby altering the tumor immune microenvironment 196.

**Figure 3:**
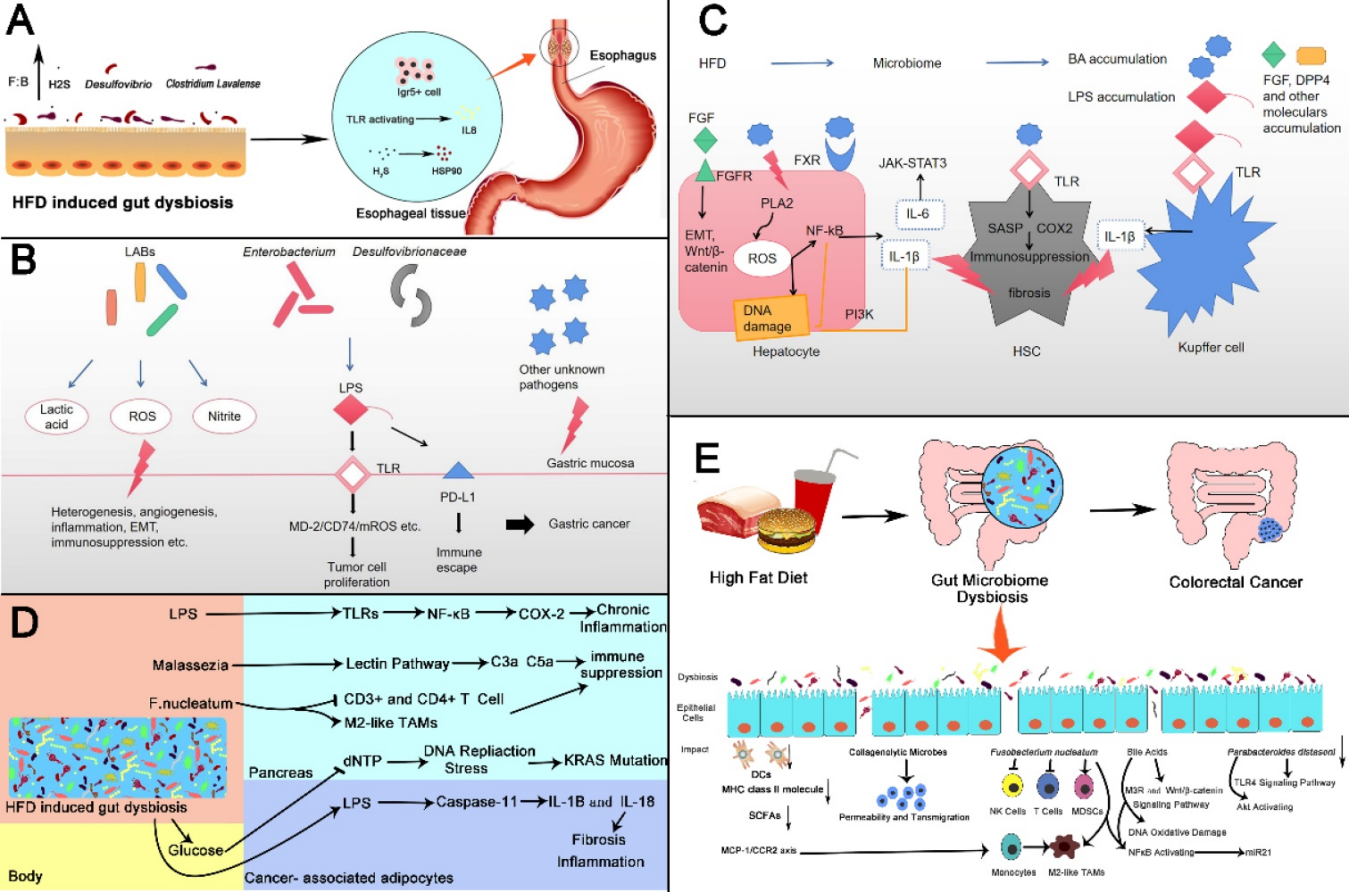
** High fat diet changes intestinal microbes and then promotes gastrointestinal cancer. (A) Esophageal Cancer**: HFD induced intestinal microbiota dybiosis (1) increases the number of Lgr5+ cells in squamocolumnar junction; (2) activates TLR signal, and promotes the activation of IL8, (3) promotes the production of H_2_S by increasing the abundance of *Desulfovibrio spp.* and *Clostridium Lavalense*. H_2_S upregulates HSP90 to promote the esophageal cancer. **(B) Gastric Cancer**: Lactic acid bacteria can produce lactic acid, reactive oxygen species, nitrite and other harmful products to promote gastric cancer in a variety of ways like heterogenesis, angiogenesis and etc. Microbiomes like *Enterobacterium* and *Desulfovibrionaceae* can stimulate TLRs and induce carcinogenic changes by producing LPS. LPS can also bind to PD-L1 to produce immune escape.** (C) Liver Cancer**: HFD changes intestinal microbial composition, leading to accumulation of certain bacterial components and metabolites. FGF and bile acids(BAs)can activate corresponding receptors on hepatocytes, trigger key oncogenic pathways then promote proliferation of cancer cells, release of inflammatory factors or DNA damage. bile acids can also activate TLR on hepatic atellate cells leading to immunosuppression. Increased LPS can activate TLR receptors on Kupffer cells to produce IL-1β and promote fibrosis** (D) Pancreatic Cancer**: HFD induced gut dysbiosis leads to the hyperglycemia, the increased LPS as well as pathogenic microorganisms such as *Malassezia* and *F. nucleatum*. These changes cause chronic inflammation, immune suppression, KRAS mutation fibrosis of pancreas through various mechanisms, which promotes the initiation and progression of cancer.** (E) Colorectal Cancer:** HFD induced gut microbiome dysbiosis including some specific microbes changes such as Collagenolytic microbes, *Fusobacterium nucleatum*, *Parabacteroides distasoni* and microbial metabolites for instance bile acids, resulting in activation of carcinogenic pathways, changes of tumor immune microenvironment, DNA damage. There also exist reduction in DCs, MHC class II molecule and SCFAs. The above changes urge the development in colorectal cancer. SCJ: Squamocolumnar junction.

Based on previous related studies, we propose other possible mechanisms. First, bile acids are one of the important metabolites of intestinal microbes 197. HFD can accelerate the increase of bile secretion induced by saturated fatty acids into the intestine 198. Gram-positive bacteria such as *Clostridium* in the intestine can convert primary bile acids synthesized by liver into secondary bile acids 199, and excessive exposure to bile acids can promote CRC 199. Second, considering the effect of HFD on intestinal microbes and the promotion of carcinogenic microorganisms on CRC, we found that, under the effect of HFD, the abundance of *Parabacteroides* in the intestine decreased 187. Some studies found that *Parabacteroides distasonis* can reduce the activation of TLR4 signaling pathway and Akt, and inhibit the promoting role of HFD on CRC. Therefore, we speculate that HFD reduces the abundance of *Parabacteroides distasonis* and indirectly promotes the development of CRC 200. Similarly, we have also found that HFD can lead to an increase in *Temperate phage* and *Fusobacterium* 201. As mentioned above, both types of microorganisms have a cancer-promoting effect. For *F. nucleatum*, it was found that the western diet has a strong correlation with *F. nucleatum*-positive CRC in an epidemiological survey, and it plays an important role in diet-mediated CRC, which further demonstrates the accuracy of our hypothesis 202. Figure 3 describes the possible mechanisms of the abovementioned cancers.

## Prospect

In the preceding narrative, we demonstrated that HFD, intestinal microbes and gastrointestinal tumors are related to each other. There is a solid theoretical basis for the suggestion that the HFD can promote the occurrence of gastrointestinal cancer by changing intestinal microbes. It can be used as a direction for future research and experiments to verify whether this proposal is correct, but in the research process, we need to pay attention to distinguish whether HFD promotes tumors directly or requires microbial mediation. In addition, because the intestinal tract and various organs are linked in many ways, even if HFD unbalances intestinal microorganisms, its cancer-promoting effect is mediated through a long-distance method or when the pathogenic microorganisms directly enter the organs. These all need further research and elaboration.

In the treatment of tumors, the relationship between HFD, intestinal microbes and gastrointestinal tumors also provides us with new ideas. First, we suggest changing the lifestyle of HFD and reduce the amount of fat in the diet to prevent the occurrence of gastrointestinal tumors, and effectively control the incidence of tumors from food-borne pathways. Secondly, for patients already suffering from disease, it is also extremely important to take effective treatment measures to prevent or even interrupt the development of tumors, and intestinal microorganisms may be an effective target. Geller and colleagues found that removing cytidine deaminase or using antibiotics such as ciprofloxacin to remove *Gammaproteobacteria* may make the tumor sensitive to gemcitabine 117. Antibiotics may be a strong candidate for future clinical trials. However, excessive exposure to antibiotics can cause dysbiosis and promote tumorigenesis 203. Therefore, there is still a need for more research on antibiotic administration methods and the feasibility of treating gastrointestinal tumors. Given that gastrointestinal tumors may have an immunosuppressive tumor microenvironment, we reason that targeted immunotherapy may also be a valuable therapeutic direction. Targeted therapy can be specifically designed based on these immune cells, their action molecules and cytokines to improve the survival rate of gastrointestinal cancer. For example, the removal of Treg can reduce the tumor burden of mouse models 204, inhibit indoleamine 2,3-dioxygenase-1 activity and enhance the tumor-specific T cell response, reduce the conversion to Treg-like cells 205. However, there is a need for a deeper understanding of tumor microenvironment components, which may make immunotherapy a more effective treatment.

## Others

Among many lifestyle factors, the diet is one of the most significant factors affecting the intestinal microbiome 206. In addition to HFD, many other lifestyle factors also have important influences on the occurrence and development of the microbiome and cancer. Human dietary habits are complex and diverse, and when studied individually, each major micronutrient and many micronutrients have been shown to alter the gut microbiome 207. In a mouse study, the chronic high-protein diet fed for 24 weeks was shown to increase intestinal permeability, cause intestinal leakage and result in changes to the β-diversity of intestinal microbes and the microbial community structure in mice 208. In another experiment in mice, the researchers reduced the dietary carbohydrate content and increased the protein content, and found that this dietary pattern can not only limit weight gain in mice, but it can also limit the occurrence and progress of breast cancer 209. In addition, high protein diets have also been shown to significantly increase the overall survival of patients with cancer 210. A high salt diet may affect gut microbial components, such as lower *Lactobacillus spp*. and change the fecal contents of short chain fatty acids such as butyrate. This can induce proinflammatory genes such as Rac1, Map2k1, Map2k6 and Atf2, as well as many cytokines and chemokines (Ccl3, Ccl4, Cxcl2, Cxcr4 and Ccr7), thereby affecting intestinal immunity and promoting inflammation 211, 212. In terms of cancer, a high-salt diet has been shown to inhibit tumor growth in mice by regulating the activity of MDSCs, activating anti-tumor immune surveillance 213. A high-sugar diet can also reduce the cecal microbial diversity 214, 215 and can be a factor that promotes the occurrence of cancer. For a high-fiber diet, researchers such as Marques found that a large amount of fiber intake can increase acetate-producing bacteria. The presence of fiber and acetate can improve intestinal imbalance 216. A high-fiber diet can extend the survival time of patients with CRC and improve the prognosis of patients with CRC. For the immune system, it regulated the activation of B and T cells to exert a positive impact on immune disorders 217, 218. Meanwhile, some micronutrients such as vitamins and zinc can also affect the composition of intestinal microbes 219, 220.

To a certain extent, the composition of the gut microbiota may depend on the individual's genetics, although studies by scholars have shown that genes play a minor influence on the formation of gut microbes 221. Khan and colleagues found immune genes regulated the selected bacterial lineages in the mouse intestine. For example, the abundance of segmented filamentous bacteria and *Mucispirillum* is related with MHC-allele-dependent IgA response 222. Some scholars have also explored the impact of host genetics on gut microbes in patients with IBD by combining whole exome sequencing and metagenomic shotgun sequencing, and found that the reduction in the levels of the microbial acetyl-CoA and glyoxylate metabolic pathways correlated with the minor allele (C) of a variant located in the gene MYRF, while the immune-related gene CABIN1 provided a more conducive environment to the growth of the *E. coli* 223. In NOD2 knock-out mice, gut microbes changed significantly. Compared with WT mice, *Alistipes (Rikenellaceae)* and *Bacteroides (Bacteroidaceae)* genera were over-represented in Nod2 knock-out mice. Conversely, the *Prevotella (Prevotellaceae)* genus was reduced 224. Researchers such as Bonder have systematically studied the impact of host genotype on gut microbes. They found multiple associations between genetic variation and the composition and function of the human gut microbiome, such as the recessive effect of an lactase functional variant on the abundance of *Bifidobacterium* 225. In a study of twins, researchers also found that the host gene ALDH1L1 was potentially related to the bacterium SHA-98, which was a component of the *Christensenellaceae* consortium 226. In general, host gene-related mutations, including single nucleotide polymorphism, gene copy number variation, non-coding RNA regulation, etc., will cause changes in host microorganisms 227. Gene-microbe interactions can also affect the occurrence of immune diseases. In the research center of Chu, H. et al., it was found that microorganisms, as an environmental factor, interact with ATG16L1/NOD2 genes to promote anti-inflammatory immune responses 228.

Molecular pathology epidemiology is also an important direction of microbial research in recent years. The tumorigenesis promoted by HFD has obvious molecular pathological characteristics. For example, we mentioned above that mice fed with HFD have higher activity of KRAS 229. Under HFD conditions, mutations in the KRAS will also promote the occurrence and development of pancreatic cancer by down-regulating the expression of the key molecule FGF21 230. In liver cancer, HFD can significantly increase the serum level of DPP4. The cascade of DPP4/CCL2/angiogenesis and the inflammatory response formed by DPP4-regulated macrophage infiltration play key roles in the progression of HFD-related HCC 231. Niku and colleagues found that Western diet accelerates the formation of colorectal adenomas, which was accompanied by the loss of APC gene heterozygosity 232. When specific pathogenic microorganisms change in the intestine, the tumors catalyzed by these pathogenic microorganisms may also have obvious molecular pathological characteristics. Hale found that in the two subtypes of deficient and proficient mismatch repair CRC, intestinal microbes are significantly different, and microbes have different roles in the two subtypes of CRC 233. *C. jejuni 81-176* infection will up-regulate two cancer-promoting signal pathways including Peroxisome proliferator-activated receptors signaling pathway and calcium signaling pathway, and promote the occurrence of CRC 234. In addition, there are some other molecular pathological epidemiology related to HFD and microbes and intestinal tumors, which was involved in the former paper.

## Figures and Tables

**Table 1:** Effects of high fat diet on intestinal microorganisms and body

No.	Specimen type	Specimen Source	Technology	Microbial composition alteration	Metabolites and other alterations	First author, year
1	Mucosal and luminal contents	C57BL/6J mice	Whole-genome shotgun sequencing, 16S rRNA gene amplification	Order level: ↑*Caudovirales* order of temperate phages; Class level: ↑*Bacteroidia*, *Bacilli* and ↑*Negativicutes*	—	Kim M.S., 2016 201
2	Fecal samples	Rat	Pyrosequencing technology, NMR	↑*Firmicutes*/*Bacteroidetes* ratio	↑Fecal tyrosine and phenylalanine. ↓Fecal amino acids, SCFAs, purines, pyrimidines, niacin, bile acids, ethanol, hexoses, N-acetyl-D-glucosamine, TCA cycle intermediates, gut microbiota related metabolites including 4-hydroxyphenylacetate, trimethylamine and dimethylamine.	Lin H., 2016 235
3	Fecal samples	C57BL/6 mice	16S rRNA sequencing	Phylum level: ↑*Firmicutes*; Family level: *Lachnospiraceae*, *Streptococcaceae* (phylum *Firmicutes*)	↑Plasma: leptin, TNFα, IL-6. Colon and ileum: inducible nitric oxide synthase (iNOS) and Ki67.	Zeng H., 2016 236
4	Fecal samples	C57BL/6J mice, 129S6/Sv mice	HiSeq-based whole genome sequencing	Genus level: ↑*Clostridium*,* Pseudoflavonifractor*, *Spirochaetes*, *Fusobacteria*, *Dorea*, *Synergistetes*, *Faecalibacterium*, *Eubacterium*, *Oscillibacter*, *Ruminococcus*, *Subdoligranulum*, *Anaerotruncu*, *Blautia*, *Euryarchaeotadramatic*, *Firmicutes*/*Bacteroidetes* ratio; ↓*Tannerella*, *Parabacteroides*, *Prevotella*	↑Genes expression involved in pathways and modules related to fatty acid metabolism, cell mobility, transport, methane metabolism, and xenobiotic degradation; capacity for glycerol utilization of the gut microbiota. ↓Genes expression involved in translation and vitamin biosynthesis.	Xiao L., 2017 187
5	Intestinal contents	C57BL/6 mice	Real-time PCR	Genus level: ↑*Firmicutes*, *Lactobacillus*; ↓*Turicibacter*, *Prevotella*, *Bacteroides*, *Bifidobacteria*.	↑Intestinal inflammatory cytokines including TNF-α, IL-1β, and IL-6. ↑Serum IFNγ and TNF-α.	Guo X, 2017 237
6	Cecal contents	C57BL/6J, 129S1/SvImJ and 129S6/SvEvTac mice	16S rRNA sequencing	Phylum level: ↑*Bacteroidetes*, *Verrucomicrobia*	↑Cecum: bile acids, AMP, cAMP, ADP, and CMP and nucleosides; plasma: proinflammatory fatty acids, such as adrenic and stearic acid. ↓Plasma: anti-inflammatory fatty acids, such as eicosopentaenoic and docosohexanoic acids.	Fujisaka S., 2018 238
7	Fecal samples	Mice	16S rRNA sequencing	Order level: ↑*Lactobacillales*; *Bacteroidale*, *Erysipelotrichales*, *Burkholderiales* (all are subject to *Bacteroidete*); Genus levels: *Lactobacillus Firmicutes*/*Bacteroidetes* ratio.	↑Serum: triglyceride, cholesterol, and high density lipoprotein; membrane transport and carbohydrate metabolism. ↓Adipose tissue: genes related to lipid metabolism expression such as PPARɑ/γ, LXRɑ/β; Liver: genes related to lipid metabolism expression such as and PPARγ and LXRɑ; metabolism of amino acid, energy, and cofactors and vitamins.	Yin J., 2018 239
8	Cecum contents	Hens	16S rRNA gene amplification, pyrosequencing	Family level: ↑*Erysipelotrichaceae*, *Alcaligenaceae*, *Enterococcaceae*, *Lactobacillaceae*, ↓*Firmicutes*/*Bacteroidetes* ratio; *Rikenellaceae*.	↑TC, TG, low-density lipoprotein cholesterol *Lactobacillaceae*, *Erysipelotrichaceae* are positively linked with LDL-C, TC, and TG. *Ruminococcaceae* had a significantly positive association with glucose. *Bacteroidaceae* and *Porphyromonadaceae* had significantly negative relationships with glucose and TG.	Liu C., 2018 240
9	Faecal samples	Young adults	16S rRNA sequencing	Genus level: ↑*Alistipes*, *Bacteroides*; *Blautia*, *Faecalibacterium Firmicutes*/*Bacteroidetes* ratio	↑Four pathways: steroid hormone biosynthesis, lysosome pathway, arachidonic acid metabolism and lipopolysaccharide biosynthesis. Faecal metabolites: indole, palmitic acid, stearic acid, arachidonic acid and indoleacetic acid; plasma:hypersensitive-c-reactive-protein and thromboxane B2; ↓Faecal metabolites: butyric acid, valeric acid and ethylmethylacetic acid.	Wan Y., 2019 241

↑: Increased; ↓: Decreased.

**Table 2:** Intestinal microbial action on cancer mechanism

No.	Types of cancer	Intestinal microbes	Molecules	Tumor promoter or suppressor	Mechanism	First author, year
1	Colon cancer	*Fusobacterium nucleatum*		Promoter	*Fusobacterium nucleatum* increases tumor multiplicity and selectively recruits tumor-infiltrating myeloid cells, which can promote tumor progression.	Kostic A.D., 2013 242
2	Colon cancer	*Fusobacterium nucleatum*		Promoter	Fap2 protein of *F. nucleatum* directly interacted with TIGIT, leading to the inhibition of NK cell cytotoxicity.	Gur C., 2015 130
3	Colon cancer	*Fusobacterium nucleatum*		Promoter	*Fusobacterium nucleatum* is inversely associated with CD3+ T-cell density in colorectal carcinoma tissue.	Mima K., 2015 188
4	Colon cancer	*Fusobacterium nucleatum*	Fap2 (fusobacterial lectin)	Promoter	Fap2 mediates attachment of *F. nucleatum* to Gal-GalNAc which is highly expressed in human CRC, metastases, and a preclinical CRC model.	Abed J., 2016 131
5	Colon cancer	*Fusobacterium nucleatum*		Promoter	*F. nucleatum* activates TLR4 signaling to MYD88, leading to activation of NFκB and increased expression of miR21; this miRNA reduces levels of the RAS GTPase RASA1.	Yang Y., 2017 243
6	Colon cancer	*Fusobacterium nucleatum*		Promoter	*Fusobacterium nucleatum* induces Annexin A1 expression in cancerous cells through FadA and E-cadherin, and FadA, E-cadherin, Annexin A1, and β-catenin form a complex.	Rubinstein M.R., 2019 133
7	Colon cancer	*Escherichia coli*		Promoter	Colibactin-producing *E. coli* contribute to the emergence of senescent cells, which enhance tumour promotion via growth factor secretion.	Cougnoux A., 2014 244
8	Colon cancer	*Escherichia coli*		Promoter	Colon cancer-associated *E. coli* bacteria induce COX-2 expression in human macrophages by p38 MAPK.	Raisch J.,2015 137
9	Colon cancer	*Bacteroides fragilis*		Promoter	ETBF-triggered colon tumorigenesis is associated with an IL-17-driven myeloid signature characterized by subversion of steady-state myelopoiesis in favor of the generation of protumoral monocytic-MDSCs.	Thiele Orberg E., 2017 141
10	Colon cancer	*Bacteroides fragilis*		Promoter	BFT triggers a pro-carcinogenic, multi-step inflflammatory cascade requiring IL-17R, NF-κB, and Stat3 signaling in colonic epithelial cells.	Chung L., 2018 140
11	Colon cancer	(1)*Lachnospiraceae bacterium A4* (2)*Helicobacter hepaticus* (3) *Mucispirillum schaedleri*		Suppressor; promoter; promoter	(1)*Lachnospiraceae bacterium A4* is related to the production of Butyrate by promoting butyrate kinase synthes. (2)*Helicobacter hepaticus* has increased RNA counts of genes involved in oxidative phosphorylation,which suggests it exerts an oncogenic effect through oxidative damage. (3) *Mucispirillum schaedleri* is increasing inflammation through increased LPS production.	Daniel S.G., 2017 127
12	Colon cancer	Commensal gut fungi		Suppressor	Commensal gut fungi mediate inflammasome activation by SYK-CARD9 Signaling Axis to restrict colon cancer.	Malik A., 2018 146
13	Colon cancer		Sirtuin-3 (Sirt3)	Suppressor	Gut microbiota (mainly *Escherichia/Shigella*, *Lactobacillus reuteri* and *Lactobacillus taiwanensis*) and Sirtuin-3 can interact with another and exert an anti-inflammatory and tumor-suppressing impact.	Zhang Y., 2018 245
14	Colon cancer	*Campylobacter jejuni*	Cytolethal distending toxin (microbial metabolites)	Promoter	*Campylobacter jejuni* promotes colorectal cancer through the genotoxic action of cytolethal distending toxin, which has DNAse activity and causes DNA double-strand breaks.	He Z., 2019 144
15	Colon cancer		P-cresol (microbial metabolites)	Promoter	Exogenous p-cresol further increased DNA damage, and independently p-cresol induced DNA damage in a dose-dependent manner against HT29 and Caco-2 cells and influenced cell cycle kinetics.	Al Hinai E.A., 2019 246
16	Colon cancer		Flagellin (microbial componments)	Promoter	Flagellin increase IL6 and CCL2/MCP-1 mRNA and IL6 excretion and cytotoxicity, decrease caspase-1 activity and the production of reactive oxygen species of CRC cells.	Pekkala S., 2019 148
17	Colon cancer liver metastasis		Lipopolysaccharide (microbial componments)	promoter	LPS promote CRC metastasis by stimulating TLR4 signaling and increasing β1 integrin-mediated cell adhesion.	Hsu R.Y., 2011 247
18	Colon cancer liver metastasis		Lipopolysaccharide (microbial componments)	Promoter	Trapping LPS reduced liver metastasis of primary CRC and attenuated metastasized tumor growth in the liver.	Song W., 2018 248
19	Gastric cancer	*Helicobacter*, intestinal commensals		Promoter	The gastric carcinoma microbiota is dysbiotic and characterised by reduced microbial diversity, reduced *Helicobacter* abundance and over-representation of bacterial genera that include intestinal commensals.The microbial community found in gastric carcinoma has increased nitrosating functions consistent with increased genotoxic potential.	Ferreira RM, 2018 249
20	Gastric cancer	*Helicobacter pylori*	P-cresol (microbial metabolites)	Promoter	*H. pylori* increase proliferation in a strain-specific manner in a novel gastroid system. *H. pylori* also alter expression and localisation of claudin-7 in gastroids and human epithelial cells, which is mediated by β-catenin and snail activation.	Wroblewski LE, 2015 250
21	Gastric cancer	*Helicobacter pylori*	CagPAI	Promoter	EMT-like morphological changes, specifically induced by cagPAI+ *H. pylori* in gastric epithelial cells, are associated to enhanced expression of mesenchymal genes and are regulated by a tripartite NF-κB/ZEB1 signaling pathway	Jessica Baud, 2013 251
22	Gastric cancer	*Helicobacter pylori*	CagA	Promoter	Degradation of p53 induced by bacterial CagA protein is mediated by host HDM2 and ARF-BP1 E3 ubiquitin ligases, while the p14ARF protein counteracts *H. pylori*-induced signalling.	Jinxiong Wei, 2015 252
23	Gastric cancer	Bacterial overgrowth and diversification		Promoter	*Lactobacillus* and *Lachnospiraceae* uncultured are enriched in GAC. The gastric microbiota is altered in patients with GAC and is correlated with bacterial overgrowth and diversification. Enrichment of microbiota potentially associated with cancerpromoting activities.	Wang, 2016 253
24	Gastric cancer	LAB, oral bacterial species	SCFA, lactic	Promoter	16S rRNA transcript sequencing *Helicobacter pylori* infection status affects overall constitution of the gastric microbiota. Increased bacterial diversity in GAC. Enrichment of proinflammatory oral bacterial species in GAC. Increased abundance of LAB and upregulated SCFAs production metabolism.	Castaño-Rodriguez, 2017 254
25	Liver cancer		Bile acids	Promoter	The altered gut microbiota causes sustained retention of high concentrations of hepatic bile acids, and then promote liver carcinogenesis.	Xie G., 2016 27
26	Liver cancer		Lipoteichoic acid (microbial componments) deoxycholic acid (microbial metabolites)	Promoter	Deoxycholic acid and lipoteichoic acid derived from the gram-positive gut microbiota cooperated to upregulate the expression of SASP factors and COX2 in DCA-induced senescent hepatic stellate cells through TLR2.	Loo T.M., 2017 172
27	Liver cancer		Bile acid	Suppressor/promoter	Primary bile acids increases CXCL16 expression, which recruits CXCR6^+^ natural killer T cells to the liver, and mediate liver tumor inhibition, whereas secondary bile acids showed the opposite effect.	Ma C., 2018 10
28	Liver cancer	SCFA-producing bacteria	SCFA (microbial metabolites)	promoter	Dietary soluble fibers are fermented by gut bacteria into SCFAs, which promotes hepatocyte proliferation, liver fibrosis and induces cholestatic liver cancer.	Singh V., 2018 255
29	Liver cancer		Interleukin-25	promoter	Dysbiosis of gut microbiota results in secretion of IL-25, which promotes the progression of HCC through inducing alternative activation and CXCL10 secretion of macrophages in tumor microenvironment.	Li Q., 2019 256
30	Breast cancer		Lithocholic acid (microbial metabolites)	Suppressor	Lithocholic acid can limit the proliferation of breast cancer cells *in vitro* and *in vivo* through activating TGR5 receptor.	Mikó E., 2018 257
31	Breast cancer	Gut microbiome		Promoter	Commensal dysbiosis promoted early inflammation within the mammary gland, enhanced fibrosis and collagen deposition both systemically and locally within the tumor microenvironment and induced significant myeloid infiltration into the mammary gland and breast tumor.	Buchta Rosean C., 2019 258
32	Breast cancer		Lithocholic acid (microbial metabolites)	Suppressor	Lithocholic acid decreases nuclear factor E2-related factor 2 expression, increases KEAP1 expression via activation of TGR5 and constitutive androstane receptor, elicits oxidative stress that slows down the proliferation of breast cancer cells.	Kovács P., 2019 259
33	Breast cancer		Cadaverine (microbial metabolites)	Suppressor	Cadaverine exerts fuctions through trace amino acid receptors to reduce breast cancer metastasis and induce a mesenchymal-to-epithelial transition and invasion.	Kovács T., 2019 260
34	Pancreatic cancer	Gut microbiome		Promoter	Gut microbiome interacts with immune system and affects cancer progression, gut microbiome depletion causes a significant anti-tumor influence in TME, such as increase in Th1 and Tc 1 cells.	Sethi V., 2018 121
35	Pancreatic cancer	*Bifidobacterium pseudolongum*		Promoter	A distinct gut microbiome was associated with immunogenic reprogramming of the PDAC tumor microenvironment. *Bifidobacterium pseudolongum* promoted mitigating M1 differentiation of macrophages.	Pushalkar S., 2018 120
36	Pancreatic cancer	*Malassezia*		Promoter	*Malassezia* acting as pathogenic fungi promote PDAC by driving the C3 complement cascade through the activation of MBL.	Aykut B., 2019 11
37	Esophagus cancer		Bile acids	Promoter	Bile acids exposed mice were easier to developed EAC and Barrett esophagus, with acute and chronic immune response, activate differential gene expression and expansion of gastric cardia progenitor cells.	Quante M., 2012 70
40	Esophagus cancer	Gut microbiome		Promoter	HFD promoted dysplasia by altering the esophageal micro-environment and gut microbiome, thereby inducing inflammation and stem cell expansion.	Münch N.S., 2019 69
41	Lung cancer		Propionate (microbial metabolites)	Suppressor	Propionate inhibited lung cancer cell proliferation by inducing cell cycle arrest, especially in the G2/M phase. It increased cleaved PARP-1 and caspase 3 expression by down- and upregulating survivin and p21.	Kim K., 2019 261
42	Melanoma	*Bifidobacterium*		Suppressor	*Bifidobacterium* showed a positive association with antitumor T cell responses within the tumor, and it promoted expression of genes associated with antitumor immunity of dendritic cells.	Sivan A., 2015 262

## Abbreviations

HFD: high fat diet
BFT: Bacteroides fragilis toxin
CDT: cytolethal distending toxin
CRC: colorectal cancer
DC: dendritic cell
EMT: epithelial-mesenchymal transition
ETBF: Enterotoxigenic Bacteroides fragilis
FadA: Fusobacterium adhesin A
FFA: free fatty acid
GAC: gastric adenocarcinoma
H_2_S: Hydrogen sulfide
HCC: hepatocellular carcinoma
INS-GAS: insulin-gastrin
LAB: lactic acid bacteria
LPS: lipopolysaccharide
MBL: mannose binding lectin
MDSC: myeloid-derived suppressor cell
NASH: Non-alcoholic steatohepatitis
PanIN: pancreatic intraepithelial neoplasms
PDAC: pancreatic ductal adenocarcinoma
PSC: pancreatic stellate cell
ROS: reactive oxygen species
SASP: senescence associated secretory phenotype
SCFA: short-chain fatty acid
SH2: Src homologous domain 2
TAM: tumor-associated macrophage
TAN: tumor-associated neutrophil
